# Calcium Phosphate–Poly(methyl methacrylate) Composite Layers Synthetized in Radio-Frequency Magnetron Sputtering Discharge

**DOI:** 10.3390/polym18050547

**Published:** 2026-02-24

**Authors:** Andreea Groza, Maria E. Hurjui, Sasa A. Yehia-Alexe, Bogdan Butoi, Silviu D. Stoica

**Affiliations:** National Institute for Lasers, Plasma and Radiation Physics, 077125 Măgurele, Ilfov, Romania; maria.zarif@inflpr.ro (M.E.H.); sasa.yehia@inflpr.ro (S.A.Y.-A.); bogdan.butoi@inflpr.ro (B.B.); daniel.stoica@inflpr.ro (S.D.S.)

**Keywords:** calcium phosphate, PMMA, radio-frequency magnetron sputtering, optical emission spectroscopy, single Langmuir probe

## Abstract

Calcium phosphate–poly(methyl-methacrylate) composite layers have been synthetized on silicon substrates in magnetron sputtering discharge by adjusting the radio-frequency power. The electron energy distribution function measured at holder substrate position shifts to lower energies when the radio-frequency power applied to the magnetron source increases from 50 to 150 W and the poly(methyl-methacrylate) molecule dissociation is augmented. The optical emission spectral analysis indicated the dynamics of the excitation and ionization processes in the Ar–calcium phosphate–poly(methyl-methacrylate) plasma mixture, as well as the dissociation patterning of the polymer molecules. The Ca I, P I, and H_α_ atomic lines and CaO, PO, POH, CO, CH and C_2_ molecular bands characteristic to the calcium phosphate and poly(methyl-methacrylate) decomposition were evidenced. At 150 W radio-frequency power a reduction in the polymer content in the composite layer volume was observed even if the α-CH_3_ main chain and the C=O molecular bands are still present. More C-C/C-H, C-OH/C-O-C polymeric bonds were revealed at the layer surface, indicating the formation of plasma polymers. The Ca/P ratio changes from 1.72 to 1.9 at 50 to 150 W, respectively, maintaining the amorphous structure of the layers. In this power range, the transition of layer surface morphologies from grain-like to worm-like plasma polymer characteristics is connected to an increase in plasma ion density and layer thickness.

## 1. Introduction

Bone tissue is composed out of an inorganic phase, mainly hydroxyapatite (HAp), an organic phase, and water [1]. The inorganic part is responsible for the stiffness of the bone [2], while type I collagen, which is the main component of the organic part, ensures the bone flexibility and the tensile strength [1].

For orthopedic applications, calcium phosphates (CaPs) have been intensively studied due to their similarities with the inorganic part of bones. However, they cannot completely mimic their properties, and that is why polymers have been considered as appropriate materials to imitate the characteristics of the organic part. Therefore, to tailor the mechanical properties, polymers can be used [1].

Poly(methyl methacrylate) (PMMA) is a synthetic polymer usually involved in the biomedical field as bone cement in joint replacement surgeries, as bone filler for cavities/defects, or as 3D scaffolds [2]. It is derived from its monomer, methyl methacrylate (MMA), through polymerization [3]. Usually less common, PMMA films were also reported in the scientific literature. Despite its biocompatibility, PMMA is a bioinert material. This raises concerns due to the lack of bonding with the surrounding tissues for orthopedic applications [2]. Among the possibilities to improve the bioactivity of PMMA, mixing with CaPs is considered an optimal solution [2].

Magnetron sputtering is a physical vapor deposition technique used for the deposition of thin films. The physical principle consists in the sputtering of a target material by high-energy particles, namely positive ions, while the ejected atoms and molecules deposit onto the substrate. Direct current (dc) sputtering is used for the deposition of metallic films, while, for dielectric (non-conductive) materials, radio-frequency magnetron sputtering (rf-MS) is required [4,5,6]. The last mentioned technique can also be used for the deposition of polymers [7]. Such polymers, obtained in a gas discharge, are called plasma polymers [8]. Plasma polymers have an increased level of crosslinking and no regular monomer units repetition, which leads to their random and complex structure when compared to the normal polymer [7]. One advantage of rf-MS deposited polymers is that no gaseous or liquid precursors are required [7]. According to Biederman H. [9], in the rf sputtering, the volatile polymer fragments resulting from the target act like plasma polymerization precursors, avoiding therefore the supply of monomer during the deposition [7]. Plasma polymerization is very complex and involves many chemical reactions in the discharge area [8]. Briefly, the collisions between electrons and the precursor molecules lead to precursor dissociation reactions and radical formation. This step is followed by radical–radical and radical–molecule reactions. More details on the processes involved in the synthesis of plasma polymers were described by Yasuda in 1985 [10].

Among the methods used for plasma diagnostics, two are of great use to evaluate the plasma polymerization process: the electrostatic probe (Langmuir probe), for the evaluation of plasma parameters such as electron temperature, density, and electron energy distribution function (f(ε)), and Optical Emission Spectroscopy (OES), which is a useful tool for a qualitative evaluation of the plasma chemistry, highlighting the dissociation and rearrangement reactions based on the identified species [10]. Various polymers such as Nylon, polypropylene, polyetherimide, polyethylene, polyimides, polyarylates, and, most commonly, fluorocarbon plasma polymers [7] have been synthetized.

In this work we report the deposition of CaP_PMMA coatings by the rf-MS technique at two working powers, 50 and 150 W. We evidenced the influence of this deposition parameter on the properties of the plasma and on the physicochemical properties of the layers. The molecular and crystallographic structures as well as the chemical composition of the films were investigated by Fourier Transform Infrared Spectroscopy (FTIR), X-ray Diffraction (XRD), X-ray photoelectron spectroscopy (XPS), and UV–Vis absorption spectroscopy. The morphology and the elemental composition were characterized by Scanning Electron Microscopy (SEM), surface profilometry, and Energy-Dispersive X-Ray spectroscopy (EDS). Optical Emission Spectroscopy and single Langmuir probe measurements were performed in order to understand the impact of increasing power on plasma processes and physicochemical and morphological properties of the coatings.

To our knowledge, no results have been reported on the deposition of CaP_PMMA layers by rf-MS technique at powers that could significantly influence the polymer chemical structure or layer morphology. Our previous works [11,12] were concentrated on the deposition of CaP-polymer layers at low rf power for keeping the chemical structure of polymers unaffected as much as possible. Moreover, the results reported in the scientific literature on the deposition of polymers by rf-MS are usually related to polymer–metal films and not on polymer–ceramic films. This incorporating plasma polymers derived from a mixed CaP_PMMA sputtering target highlights the novelty of the present study.

## 2. Materials and Methods

### 2.1. Materials

Silicon substrates with mirror-like surfaces of 0.5 mm thickness and cut in 10 × 10 mm^2^ pieces have been involved in the deposition of thin layers.

Calcium phosphate and poly(methyl methacrylate) powders were used for sputtering target preparation and as precursors for layers synthesis in magnetron plasma discharge.

Calcium phosphate powder (Ca_10_(OH)_2_(PO4)_6_) has the molecular weight of 1004.67 g/mol and a theoretical Ca/P ratio of 1.67 (Alfa Aesar company, Massachusetts, MA, USA, CAS number 12167-74-7).

Poly(methyl methacrylate) powder [CH_2_C(CH_3_)(CO_2_CH_3_)]_n_ has an average molecular weight of 350.000 g/mol (Sigma-Aldrich, Missouri, MO, USA, CAS number 9011-14-7).

### 2.2. Synthesis of Sputtering Target

The calcium phosphate powder (CaP) was mechanically mixed with poly(methyl methacrylate) powder (PMMA) in 90–10% percentages and the resulting compound was involved in sputtering target production. The sputtering target of 50 mm diameter and 3 mm thickness was obtained after the mechanical pressing of the CaP_PMMA powder.

### 2.3. Deposition Technique

CaP_PMMA layers were deposited on silicon substrates by magnetron sputtering technique in Ar gas flow by using a magnetron plasma source (purchased from K.J.Lesker company, Dresden, Germany) coupled to a radio-frequency (rf) power supply (CESAR rf power generator, Dressler company, Meckenheim, Germania) working at 13.56 MHz. The magnetron plasma source head has a diameter of 50 mm. The layers were deposited at 50 and 150 W rf power, 1.4 × 10^−2^ mbar working pressure, 7 mL/min Ar gas flow, and 40 mm distance between magnetron plasma source head and substrate holder. The substrate holder was grounded and the deposition time was 10 h. The deposition rate was measured by a quartz crystal microbalance (INFICON Holding AG Company, Dresden, Germany) and it was about 0.2 Å/s at 50 W and 3 Å/s at 150 W. The deposition rates indicate the thickness of the CaP-PMMA layer deposited at 50 W of about 720 nm (CaP_PMMA_50) and of that deposited at 150 W of about 10 µm (CaP_PMMA_150). More details about the magnetron sputtering deposition technique and the experimental set-up can be found in [11,12].

### 2.4. Characterization Technique

The electrical properties of the plasma generated during the deposition of CaP_PMMA layers have been investigated with the help of an ESPion Langmuir probe system (Hiden Analytical, Warrington, UK) that measures the current-voltage (IV) characteristic. The Langmuir probe is integrated in a complete fixed system that comprises an interface unit (EPIU—ESPION Probe Interface Unit), a gas-cooled, radio-frequency electrostatically compensated probe and connection cables. During the measurements, the probe current can vary in the range of 1 mA–1 A, when the voltage applied to the probe tip is swept between −150 and 100 V. The tip of the probe consists of a tungsten wire of 0.15 mm in diameter and 10 mm length for minimal plasma disturbances [13]. The Langmuir probe system is operated via computer control by running the Hiden Analytical Limited’s ESP 7.23.0.24 software.

The Langmuir probe tip is placed in the plasma at a distance of 40 mm from the magnetron plasma source head, instead of the substrate holder. Thus, the plasma properties at the substrate position, during the CaP-PMMA layer depositions, are investigated. The standard working procedure of the ESPion Langmuir probe system implies firstly the cleaning of the probe tip for 20 ms by applying a negative voltage of −100 V followed by 90 ms of recording the plasma IV characteristics. Thereby, tip probe contamination with plasma components, mainly carbon and oxygen species, is avoided. For assuring the data accuracy and repeatability, 10 measurements were performed for each IV characteristic. The IV characteristics signals recorded in the −80–80 V domain allowed calculation with high accuracy of the electron temperature, electron density and ion density, following the formulas [14]:

T_e_ (eV) = 1/α (1)

where α is the slope of the plot of natural logarithm of the electron current intensity versus the probe potential

(2) $$n_{e}=3.73\times 10^{13}\frac{I_{est}\left(A\right)}{A_{p}(m^{2})\sqrt{T_{e}(eV)}}$$

(3) $$n_{i}=\frac{1.42\times 10^{15}\;M{\left(amu\right)}^{1/2}{\left(-slope\right)}^{1/2}}{A_{p}(m^{2})}$$

where I_est_ is the intensity of the electron saturation current, A_p_ is the area of the probe tip exposed to the plasma, T_e_ is the electron temperature, and M is the mass of the ions.

The electron energy distribution function (f(ε)) as a function of probe voltage is also calculated by the ESP software by using the formula [14]:

(4) $$f(\varepsilon )=\frac{-4}{e^{2}A_{p}}({\frac{m_{e}(V_{p}-V)}{2e})}^{1/2}\frac{d^{2}I_{e}}{dV^{2}}$$

where *V_p_* is plasma potential, *V* is the probe electrode voltage, *e* is the electron charge, *A_p_* is the area of the probe, *m_e_* is the electron mass, and *d*^2^*I_e_/dV*^2^ is derived from the IV Langmuir probe characteristic.

The ionized and excited species encountered in the plasma were analyzed using an Ocean Optics (Ostfildern, Germany) model Flame-S miniature spectrometer with a resolution of 1 nm, integration time of 1 ms, 400–700 nm spectral range, and entrance slit of 25 µm.

The chemical bond state at the surface of CaP_PMMA layers was evaluated by X-ray photoelectron spectroscopy (XPS) using a K-Alpha Thermo Scientific (ESCALAB™ XI+, East Grinstead, UK) spectrometer equipped with a 180° double focusing hemispherical analyzer. Pass energies of 50 eV and 20 eV with a 0.1 eV energy step were set for survey and high-resolution spectra recording. The C 1s peak is positioned at 284.6 eV. The peak fitting analysis of the high-resolution spectra were performed by using Magic plot 2.9. software.

UV–vis spectrometer (Shimadzu UV-2600i, Kyoto, Japan) was used in a diffuse reflectance mode to measure optical absorption spectra in the spectral range of 190–450 nm.

The molecular structure of CaP_PMMA layers and the interconnectivity between CaP and polymer structure have been studied by using a Perkin Elmer SP-100 spectrometer (Waltham, Massachusetts, MS, USA), 4000–400 cm^−1^ spectral range, working in Attenuated Total Reflection (ATR) mode. Each spectrum of the CaP_PMMA sample was recorded after 32 scans. The peak fitting analysis of the IR spectra of CaP_PMMA layers was performed in 1100–900 cm^−1^ and 1800–1100 cm^−1^ spectral ranges by using the curve fitting procedure described in [15].

The structure of the CaP_PMMA powder and layers was investigated with an X-ray computerized diffractometer that possesses a Cu-Kα X-ray source with emission on 0.154 nm in a Bragg–Brentano configuration. The diffractograms were recorded in the 10°−60° angle range with an accuracy of 0.020 for 36 h.

The surface topography of the samples was analyzed with a contact profilometer (P-7 Stylus Profiler, KLA, Milpitas, California, CA, USA) by scanning 700 × 700 µm^2^ areas. The surface roughness was measured in accordance with ISO 25178 standards [16,17]. The instrument was equipped with a diamond stylus tip of 0.15 µm radius. Adjacent profiles were acquired with a lateral spacing of 10 µm, employing a scanning speed of 100 µm/s and a sampling frequency of 200 Hz. The data were processed using APEX 3D BASIC software (Version 7, Digital Surf, Besançon, France, 2016).

The surface morphology and elemental composition of CaP_PMMA layers were investigated by SEM and EDS using an FEI Inspect S scanning electron microscope (Hillsboro, Oregon, OR, USA), in both high- and low-vacuum modes, and an EDAX Inc. SiLi detector (Hillsboro, Oregon, OR, USA) for elemental compositional analysis. The 3D surface plots of the SEM images were performed by using the ImageJ 1.53e. software.

## 3. Results and Discussion

### 3.1. Electric Characterization of Plasma Discharge by Langmuir Probe Measurements

The synthesis plasma parameters of CaP_PMMA layers were investigated by using the ESPIon Langmuir probe that acquires the current-voltage characteristics (IV), further involved in the calculation of electron temperature (*T_e_*), electron density (*n_e_*), ion density (*n_i_*), and f(ε). The calculation method of these parameters was detailed in Section 2.4 and in the Hiden’s ESPion probe technique manual [13].

In Table 1 the *T_e_*, *n_e_*, and *n_i_* parameters of the magnetron plasma discharge at 50 and 150 W rf power are presented. The measurements were performed by placing the Langmuir probe tip in the plasma bulk at two different positions: (1) in the center of the plasma relative to the magnetron plasma source head (0 mm position) and (2) at 20 mm radial to the center of the plasma. For reaching this last position, the Langmuir probe was displaced by linear translation with 20 mm from the center of the plasma. There were no significant differences observed between T_e_ calculated at the two positions of the probe relative to the plasma bulk. Besides that, the n_e_ increases and n_i_ decreases when the probe moves towards the outside of the plasma bulk. During the plasma discharge, when the rf power increases, the T_e_ and n_i_ increases also, while n_e_ decreases in accordance with Formulas (3) and (2).

**Figure 1:**
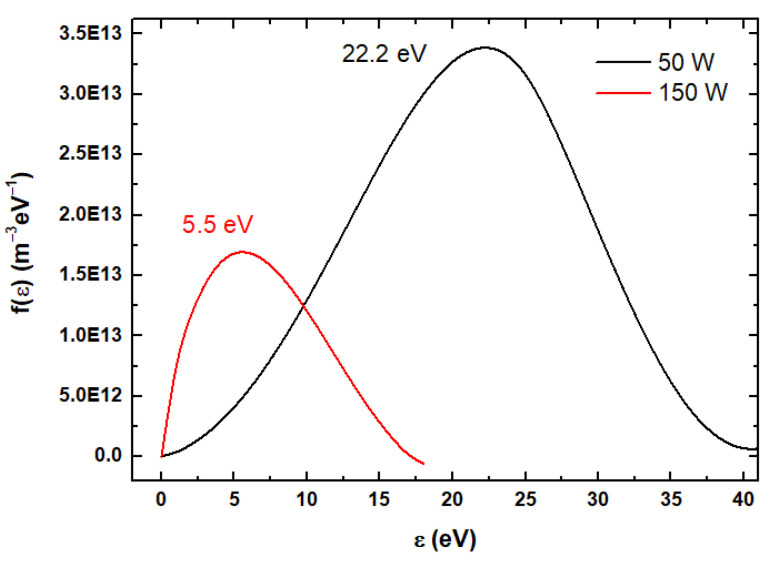
f(ε) of CaP_PMMA magnetron plasma at 50 (black line) and 150 W (red line).

In Figure 1 the electron energy distribution function (f(ε)) of the magnetron plasma discharge at 50 and 150 W rf power when the Langmuir probe was placed in the center of the plasma is illustrated. According to Hiden’s ESPion probe technique the f(ε) is calculated by using the Druyvestyn method [13]. The f(ε) is shifted towards lower energy for higher rf power (see Figure 1) as the electrons are mainly involved in the dissociation processes of PMMA compound [10] and consume a major part of their energy. Previously, it was shown that an increase in the collision rate can lead to f(ε) shift to lower energy [17,18]. Thereby, in accordance with Table 1, at 150 W rf power, more ions are generated and consecutively a higher collision rate is promoted. Therefore, the shift of f(ε) from higher energies (black line—50 W) to lower energies (red line—150 W) can be attributed to increasing plasma excitation and ionization processes.

### 3.2. Optical Emission Spectroscopy Analysis of Magnetron Plasma Discharge

**Figure 2:**
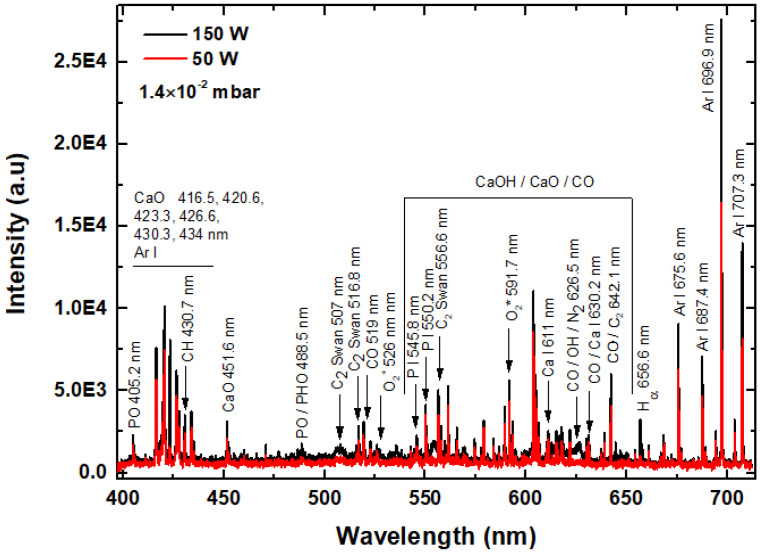
Optical emission spectra of the magnetron sputtering plasma discharge.

In Figure 2 the optical emission spectra of magnetron-sputtering plasma discharge at both 50 and 150 W rf power are illustrated. As the Ar is the working gas, the emission spectra are dominated by the atomic emission Ar I lines from the red region (4p-4s transition) at 675.6, 687.4, 696.9, and 707.3 nm and blue region (5p-4s transition) in the range of 400–450 nm [19,20]. The blue Ar I atomic lines are superposed on CaO molecular bands from 416.5, 420.6, 423.3, 426.6, 430.3, and 434 nm [21]. Other CaO molecular bands can be identified at 451.6 nm and in the wavelength range of 550–650 nm, where they superpose to the CaOH and CO molecular bands. Emission characteristics of PO molecular bands can be identified at 405.2 nm and at 488.5 nm. At 488.5 nm some molecular bands characteristic either to PO or PHO can be observed [21]. Atomic emission lines of Ca I and P I are assigned to 611 and 630.2 nm and 545.8 and 550.2 nm, respectively [22,23]. The Ca I emission line at 630 nm is superposed on CO molecular band.

The PMMA compound sputtered from the target is dissociated in the plasma, and the dissociation pattern is revealed in the optical emission spectra from Figure 2 by the identification of CH molecular band at 430.7 nm, C_2_ Swan molecular bands at 507, 516.8, 556.6, and 642.1 nm, CO molecular bands at 519, 626.5, 630.2, and 642.1 nm, O_2_ molecular bands at 526 and 591.7 nm, and H_α_ atomic line at 656.6 nm. The molecular band identified at 626.5 nm can be attributed to OH emissions and the molecular band at 642.1 nm can be assigned to CO or C_2_ according to [10,13,21,24].

Spectral intensities increase with the increase in the applied rf power indicating that, at 150 W, higher decomposition of PMMA compound in the plasma discharge takes place. In the optical emission spectra, recorded at 150 W rf power, the appearance of molecular bands of C_2_ Swan at 507 nm, CO/OH at 626.5 nm and H_α_ atomic line at 656.6 nm is highlighted. Their observation is related to the higher collision rate of electrons with the molecules in the plasma, as was pointed out by the electron energy distribution function shift to lower energies (see Figure 1). The dissociation energy of polymer bonds such as C-C, C-H/C-O and C=O are 3.5 eV, 11 eV and 7.7, respectively [11]. The excitation of these species for their spectral emission evidence requires the molecules’ collision with electrons of the following energies: 2.5 eV for C_2_ Swan, 6–11 eV for CO, and 10.2 eV for H_α_ [19,25]. Accordingly, the plasma generated at 150 W has the required parameters for breaking and excitation of the C_2_, CO, and H species with a higher probability than the plasma generated at 50 W.

### 3.3. X-Ray Photoelectron Spectroscopy

**Figure 3:**
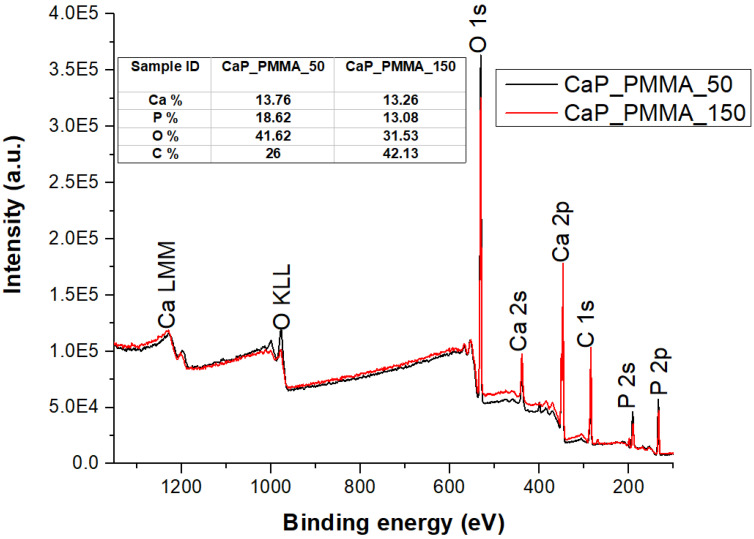
XPS survey spectra of CaP_PMMA_50 and CaP_PMMA_150 layers.

Figure 3 shows the XPS survey spectra of CaP_PMMA_50 and CaP_PMMA_150 layers. Chemical elements characteristic for CaP, Ca, P, and O, and those for PMMA, C and O, were identified in the spectra. Higher C % (42.13%) and lower O % (31.53%) contents were measured at the surface of the layer deposited at 150 W than at 50 W (26% C and 41.62% O), due to enhanced polymer decomposition at higher working power without recovery on the substrate. The reduced O content in the polymeric layers grown in plasma was previously attributed to formation of cross-linked bonds [26]. Such polymers were rich in carbon bonds and the coatings more compact and denser [26]. In this regard, to evaluate the chemical structure of the coatings, especially the presence of the polymer at the surface, high-resolution XPS spectra of C 1s and O 1s were acquired and a peak fitting analysis was conducted.

**Figure 4:**
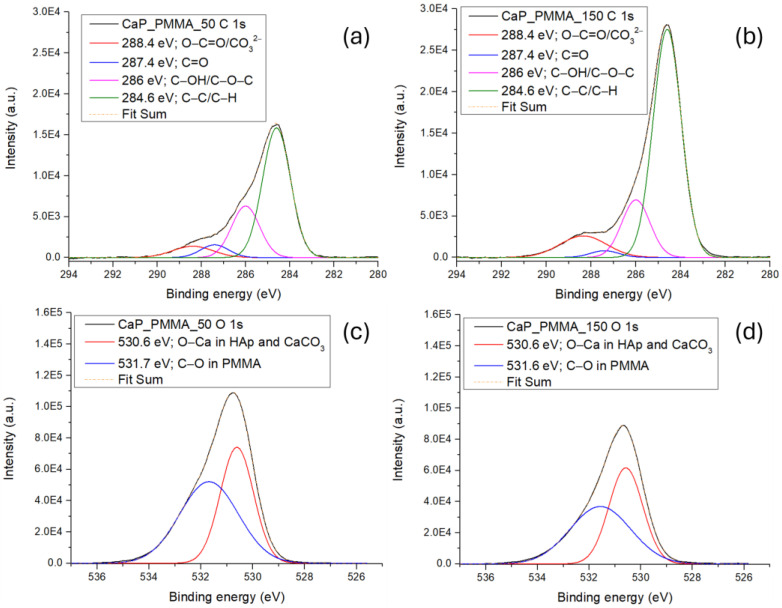
XPS high-resolution deconvoluted spectra of CaP_PMMA_50 (**a**,**c**) and CaP_PMMA_150 (**b**,**d**) of C 1s and O 1s.

Figure 4 presents the high-resolution deconvoluted spectra of C 1s (a and b) and O 1s (c and d) for the CaP_PMMA_50 and CaP_PMMA_150 layers. For the C 1s high-resolution spectra, the peaks resulting from the peak fitting analysis at 284.6 eV, 286 eV, 287.4 eV, and 288.4 eV were assigned to C-C/C-H, C-OH/C-O-C, C=O, and O-C=O/CO_3_^2−^, respectively [27,28]. The intensities of the C 1s subpeaks corresponding to the CaP_PMMA_150 layer (Figure 4b) are higher than those corresponding to the CaP_PMMA_50 layer (Figure 4a), except for the peak assigned to C=O from 287.4 eV, which is lower.

The O 1s peak-fitting analysis (Figure 4c,d) indicated the presence of two subpeaks at 530.6 eV, assigned to O-Ca in HAp and/or CaCO_3_, and at 531.6 eV/531.7 eV, assigned to C-O bonds in PMMA [29,30,31,32]. These results are in agreement with the OES investigation (Figure 2), which revealed a higher decomposition of PMMA at 150 W, and with the FTIR investigation (Section 3.5.2), which showed a closer similarity between the molecular structure of pristine PMMA and that of the CaP_PMMA_50 layer than that of the CaP_PMMA_150 layer.

### 3.4. UV–Vis Absorbance Spectroscopy

**Figure 5:**
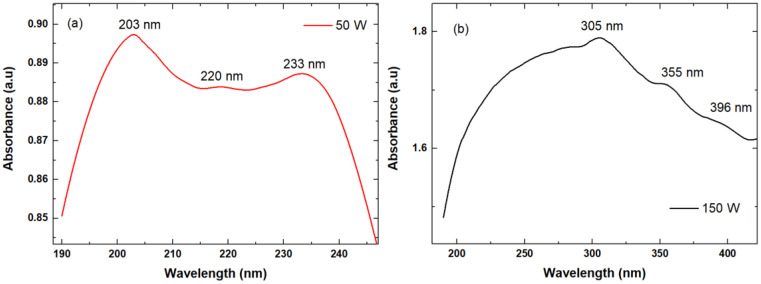
UV–Vis spectra of: (**a**) CaP_PMMA_50 and (**b**) CaP_PMMA_150 samples.

In Figure 5 the absorption spectra in the UV–Vis domain of CaP_PMMA_50 and CaP_PMMA_150 layers are illustrated. The UV–Vis absorption spectrum of CaP_PMMA_50 layer indicates three maxima-absorption wavelengths at 203, 220, and 233 nm. In accordance with refs. [33,34,35] related to these maxima absorption wavelengths and the chemical composition of calcium phosphate, the peak from 203 nm can be attributed to both Ca(OH)_2_ [36] and PO_4_ [37] and the ones from 220 and 233 nm to C-C bound from the PMMA compound [38,39]. The UV–Vis absorption spectrum of CaP_PMMA_150 layer indicates a broad absorption band between 190 and 450 nm with three maxima-absorption wavelengths at 305, 355, and 396 nm. In accordance with refs. [33,34,36,39,40,41,42] the peak from 200 nm is characteristic to CaP compound, while the peaks between 220 and 400 nm to C-C and C=O bonds characteristic to PMMA compound. However, according to UV–Vis analysis, the CaP_PMMA_150 layer indicates more evidence of PMMA compound by the presence of carbonyl group through the absorption peaks.

### 3.5. Fourier Transform Infrared Spectroscopy

#### 3.5.1. CaP-PMMA and PMMA Powders

**Figure 6:**
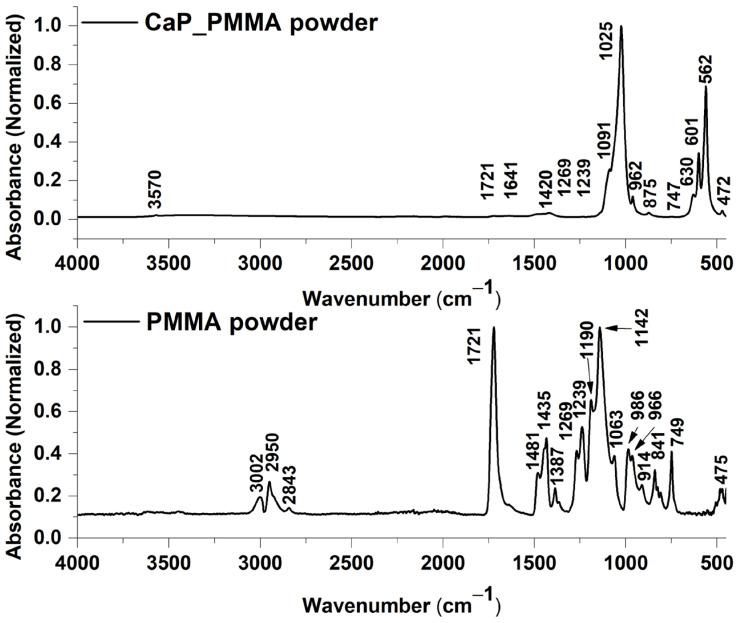
FTIR spectra of CaP_PMMA and PMMA powders.

Figure 6 presents the FTIR spectra of the CaP_PMMA and PMMA powders. The IR bands specific to PMMA are summarized in Table 2.

The bands identified in the FTIR spectrum of the CaP_PMMA powder are characteristic both to CaPs and PMMA (see Figure 6). The bands at 3570 cm^−1^, 1641 cm^−1^, and 630 cm^−1^ are characteristic for the hydroxyl group, stretching mode of lattice water, bending mode of adsorbed water, and the liberation mode (ν_L_), respectively [12]. The bands characteristic for the vibrations of the phosphate group were identified at 1091 cm^−1^ and 1025 cm^−1^ for the asymmetric stretching mode (ν_3_), 962 cm^−1^ for the symmetric stretching mode (ν_1_), 601 cm^−1^ and 562 cm^−1^ for the bending mode (ν_4_), and 472 cm^−1^ for the bending mode (ν_2_) [12,43]. The carbonate group was evidenced by the presence of the 1420 cm^−1^ band, characteristic for B-type carbonation [44], and the band positioned at 875 cm^−1^, characteristic for AB-type carbonation [43].

Besides the IR bands specific to CaP, in the FTIR spectrum of the CaP_PMMA powder the following bands were observed: 1721, 1269, and 1239 cm^−1^, corresponding to the stretching of -C=O [45,46] and C-C-O [45] and asymmetric stretching of C-O-C in the PMMA polymer [46].

**Table 2:** The assignment of the molecular bands for the PMMA powder in the 4000–750 cm^−1^ [45,46,47,48].

Wavenumber (cm^−1^)	Band Assignment	Ref.	Wavenumber (cm^−1^)	Band Assignment	Ref.
**3002**	C-H, symmetric stretching in O-CH_3_	[45,48]	**1239**	C-C-O symmetric stretching/C-O-C asymmetric stretching	[47,49]
**2950**	C-H, asymmetric stretching in O-CH_3_	[45,48]	**1190**	C-O-C/CH3 wagging	[46,50]
**2843**	CH_3_	[45]	**1142**	C-O-C, symmetric stretching	[45,46]
**1721**	C=O, stretching	[45,46]	**1063**	C-C skeletal, rocking	[45,46]
**1481**	α-CH_3_ main chain	[45]	**986**	C-O-C, rocking	[45,46]
**1447**	CH_2_, main chain, bending	[45,46]	**966**	a-CH_3_ main chain	[45]
**1435**	O-CH_3_	[45]	**912**	C-O-C	[47]
**1387**	α-CH_3_ main chain	[45]	**841**	CH_2_ skeletal	[45]
**1269**	C-C-O	[45]	**749**	CH_2_ skeletal	[45]

#### 3.5.2. CaP—PMMA Composite Layers

**Figure 7:**
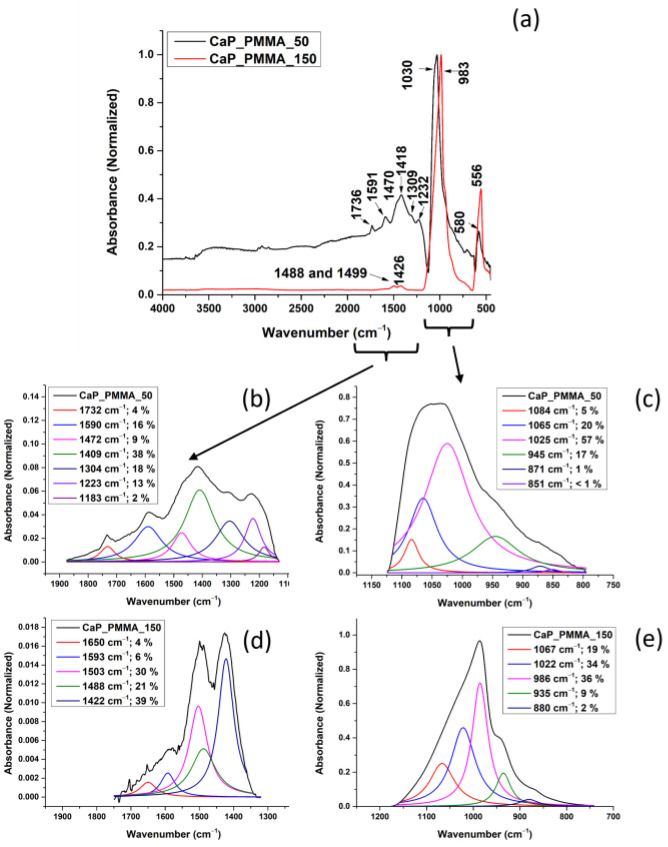
(**a**) FTIR spectra of CaP_PMMA layers deposited at 50 W and 150 W; deconvoluted FTIR spectra of CaP_PMMA_50 (**b**,**c**) and CaP_PMMA_150 layers (**d**,**e**) in the 1900–1100 cm^−1^ and 1200–750 cm^−1^ wavenumber ranges.

Figure 7a presents the FTIR spectra of the CaP_PMMA layers deposited at 50 and 150 W rf power.

In the FTIR spectrum of the layer deposited at 50 W (see Figure 7a—black line) the molecular bands characteristic to the PMMA for C=O stretching [50], C=O [51], C-O-C [52], and C-C-O symmetric stretching [48] or C-O-C asymmetric stretching [46] were identified at 1736 cm^−1^, 1591 cm^−1^, 1309 cm^−1^, and 1232 cm^−1^. The bands at 1470 cm^−1^ and 1418 cm^−1^ can be assigned to the carbonate group, indicating the B-type carbonation [44].

In the FTIR spectrum of the layer deposited at 150 W, only the band at 1488 cm^−1^ could be assigned to PMMA, more explicitly to the α-CH_3_ main chain [45]. The shift from 1481 cm^−1^ indicates changes in the molecular structure of the polymer. The band at 1426 cm^−1^ shifted from 1420 cm^−1^ (see Figure 7a—red line) can be assigned to the carbonate group [44].

Differences were also observed between the band characteristics for the phosphate group. For the CaP_PMMA layer deposited at 50 W, the band characteristic for the asymmetric stretching mode of PO_4_^3−^ slightly shifted from 1025 to 1030 cm^−1^. However, at a working power of 150 W, the band is shifted to 983 cm^−1^. This indicates a significant change in the molecular structure of the CaP layer when compared to the target material (see Figure 6) and most probably an increase in the tetra calcium phosphate (TTCP) phase in the layer [53]. This assumption is also sustained by the EDS measurements, as the Ca/P ratio for this layer increased to 1.9, which is close to 2, the Ca/P ratio of TTCP (Section 3.8). There is also the possibility that, during the CaP_PMMA layer growth on the Si substrate, the formation of P-O-C bonds happens. In a previous spectral study on organophosphorus compounds, it has been shown that P-O-C bonds can manifest their vibrations in the spectral region of 1060–850 cm^−1^ and need to be accompanied by C-O bond vibrations [54,55].

Considering the above assumptions on the 983 cm^−1^ band assignment and the fact that the FTIR bands in the wavenumber range of 1200–750 cm^−1^ and 1900–1100 cm^−1^ are broad, a peak fitting analysis was required to highlight the molecular changes generated by an increased working power and the polymer presence in the layers.

#### 3.5.3. Peak Fitting Analysis of the FTIR Spectra of CaP_PMMA Layers

The peak fitting analysis results are presented in Figure 7b–e and Table 3. In the wavenumber range 1200–750 cm^−1^, significant differences were observed between the layers deposited at 50 and 150 W.

In the deconvoluted spectra from Figure 7c,e, a decrease in the area percentage from 57% to 34% was observed for the band at 1025 cm^−1^, corresponding to the asymmetric stretching mode (ν_3_) of PO_4_^3−^, as the working power increased from 50 W to 150 W. This result, in association with those observed in the case of the absorption band at 945 cm^−1^ (17%) for the CaP_PMMA_50 layer, (characteristic for ν_1_ PO_4_^3−^ in HAp [12]) and the band at 986 cm^−1^ (36%) for the CaP_PMMA_150 layer (characteristic either to ν_1_ PO_4_^3−^ in TTCP [53] or to P-O-C bond formation [54,55]), sustains the assumption that an increase in the working power increases the amount of TTCP in the layer or promotes interconnections between CaP and polymer.

A difference between the layers was observed also in the deconvoluted spectra based on the type of carbonation that occurred during the rf-MS deposition: B-type for the layer deposited at 50 W, with an absorption band at 871 cm^−1^ [44], and A-type for the layer deposited at 150 W, with an absorption band at 880 cm^−1^. However, the carbonation was not significant, considering that the percentages associated to the peak areas were around 1–2%.

The absorption band at 1065 cm^−1^ could be assigned to the C-O-C asymmetric stretching [48] in PMMA (see Figure 7c,e). The percent assigned to the absorption band at 1065 cm^−1^ is around 20% for both deposition conditions.

For the layer deposited at 50 W, in the wavenumber range 1900–1100 cm^−1^, deconvoluted absorption bands specific to the polymer were identified at 1732 cm^−1^ (peak area percentage of 4%) and 1183 cm^−1^ (peak area percentage of 2%) and attributed to C=O stretching [50] and C-O-C vibrations [45]. The band at 1304 cm^−1^ (peak area percentage of 18%) was reported in the literature as C-O-C in the MMA monomer (see Table 3), indicating, most probably, the partial decomposition of PMMA. As the working power was increased, only the band at 1650 cm^−1^, characteristic for C=O symmetric stretching [56], was identified after the peak fitting analysis (see Figure 7d). An increased number of C-O-C bonds was identified at the surface of CaP_PMMA_150 sample in accordance with the XPS spectra of C1s peak from Figure 4b.

The deconvoluted absorption bands at 1472 and 1409 cm^−1^ indicate a B-type carbonation in the layer deposited at 50 W, while the deconvoluted absorption bands at 1503 and 1422 cm^−1^ indicate both A-type and B-type carbonation at 150 W rf power [44]. Despite the fact that the peak area percentages associated with the carbonate group are higher for the layer deposited at 150 W, no assumption on the carbonation degree can be made considering that the intensities of the bands are much lower than those in the deconvoluted FTIR spectra of the layer deposited at 50 W.

In the OES spectra from Figure 2 CH, CO, C_2,_ PO and PHO molecular bands were identified due to the sputtering of CaP_PMMA target and further dissociation of CO_3_^2−^, -CH_3_ -CH_2_ and PO_4_^3−^ groups in the plasma. Their recombination on the substrate surface led to the growth of a layer and therefore the CO_3_^2−^, -CH_3_, C-O, C=O or PO_4_^3−^ groups were identified in their FTIR spectra (see Table 2 and Table 3). Increasing the rf power up to 150 W, the intensity of carbon-based species emission in the plasma grows, indicating a higher degree of sputtering and decomposition of CaP and PMMA. The C_2_ Swan band from 507 nm, CO/OH bands from 626.5 nm and H_α_ atomic line from 656.6 nm are present only in the OES spectrum of plasma produced at 150 W, due to enhanced dissociation processes, explaining the reduced presence of C=O, C-O-C, C-C-O and CH_3_ bands in the FTIR spectrum of CaP_PMMA_150 (see Figure 7). Once the C=O, C-O-C, C-C-O and CH_3_ bonds are broken by the energetic electrons and not recovered in the layer, their intensities in the FTIR spectra are reduced and visible only after peak fitting analysis. On the other hand, the intensity of the PO_4_^3−^ band from 986 cm^−1^ found in the FTIR spectrum of CaP_PMMA_150 layer increases (see Figure 7). The XPS C1s, high-resolution spectra showed more C-C/C-H and C-OH/C-O-C bonds at the surface of the Cap_PMMA_150 layer.

**Table 3:** The assignment of the deconvoluted FTIR absorption bands for the CaP_PMMA deposited at 50 and 150 W in the wavenumber ranges 1200–750 cm^−1^ and 1900–1100 cm^−1^.

CaP_PMMA 50 W			CaP_PMMA 150 W		
**Wavenumber** **(cm^−1^)**	**Band Assignment**	Ref.	Wavenumber (cm^−1^)	Band Assignment	Ref.
**1200–750 cm^−1^**					
1084	ν_1_ of PO_4_^3−^	[57]	-	-	-
1065	C-O-C asymmetric stretching	[48]	1067	C-O-C asymmetric stretching	[48]
1025	ν_3_ of PO_4_^3−^	[57]	1022	ν_3_ of PO_4_^3−^	[57]
-	-	-	986	ν_1_ of PO_4_^3−^ in TTCP P-O-C vibration	[54,55,56]
945	ν_1_ of PO_4_^3−^ in HAp	[12]	935	ν_1_ of PO_4_^3−^	[11]
871	B-type carbonation	[44]	880	A-type carbonation	[44]
		**1900–1100 cm^−1^**			
1732	C=O stretching	[50]	1650	C=O symmetric stretching	[56]
1590	C=O vibrations	[51]	1593	C=O vibrations	[51]
1472	CO_3_^2−^—B-type carbonation	[44]	1503	CO_3_^2−^—A-type carbonation	[44]
1409	CO_3_^2−^—B-type carbonation	[44]	1488	α-CH_3_ main chain	[45]
1304	C-O-C in MMA	[52]	1422	CO_3_^2−^—B-type carbonation	[44]
1223	C-O stretch	[51]			
1183	C-O-C	[45]			

### 3.6. X-Ray Diffraction

**Figure 8:**
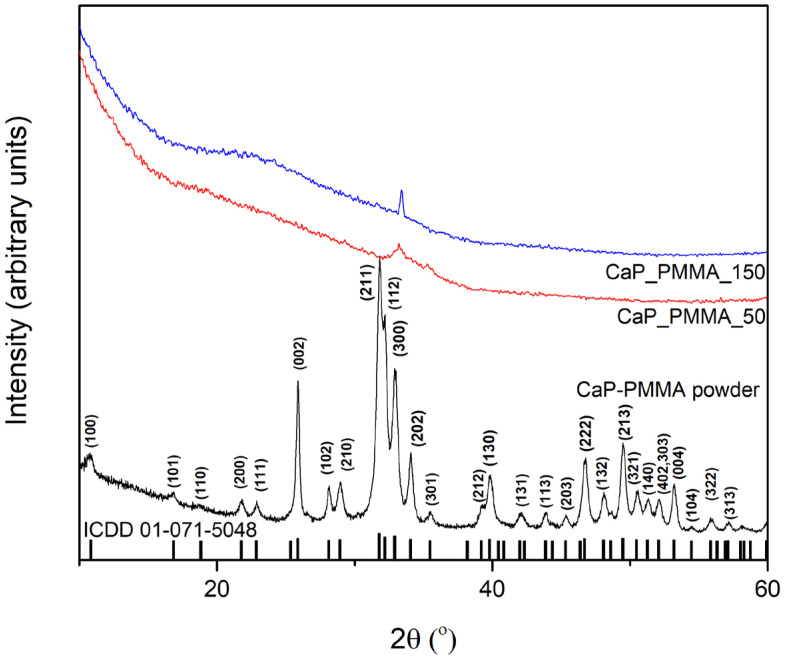
XRD pattern of CaP_PMMA powder; CaP_PMMA_50 layer; and CaP_PMMA 150 layer.

XRD pattern of CaP-PMMA powder and layers are presented in Figure 8. The amorphous structure of the layers can be observed. The diffraction peaks of the powder are in agreement with the standard card of HAp (ICDD 01-071-5048).

### 3.7. Surface Profilometry

**Figure 9:**
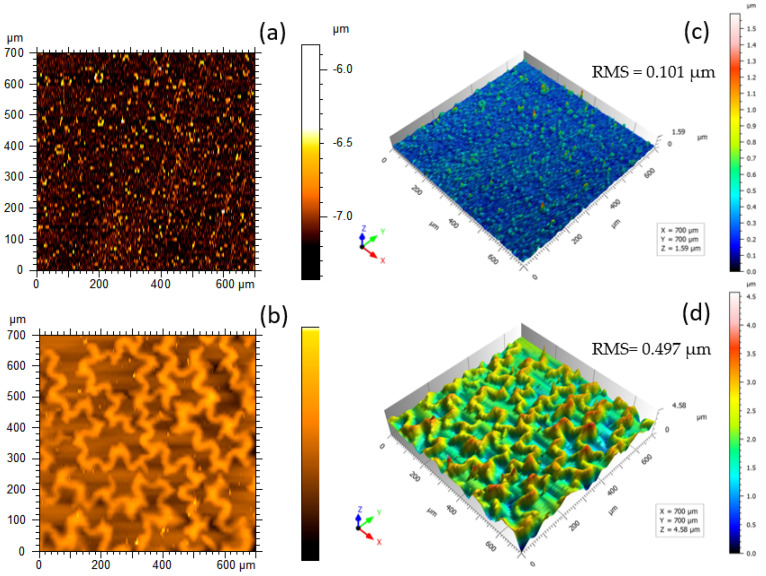
2D and 3D surface profiling of CaP_PMMA_50 (**a**,**c**) and CaP_PMMA_150 (**b**,**d**) layers.

On a micrometer scale, the 2D and 3D surface profile features of CaP_PMMA layers are revealed in Figure 9. The root mean square of the surface height deviations from the mean plane (RMS) increases from 0.101 µm (Figure 9c) to 0.497 µm (Figure 9d). The rise in RMS values indicates a more irregular geometry of the layer surface topography and consecutively an increase in surface patterning.

The images from Figure 9c,d show the growth of grain-like structures on the surface of the CaP_PMMA_50 layer and of worm-like structures on the surface of CaP_PMMA_150 layer. These worm-like structures indicate the wrinkle of the surface due to the polymer heating as the result of surface bombardment with a higher number of ions (see Table 1), atoms, and molecules. In a magnetron plasma, usually, the temperature at the substrate could attain values of 200 °C [58], thus encouraging the polymer texturizing (PMMA melting temperature ~160 °C). Such patterns were previously observed on the surface of polymer layers produced by plasma methods and attributed to the charge distribution essential in the initial phase of the polymerization process [59].

### 3.8. Scanning Electron Microscopy and Energy-Dispersive X-Ray Spectroscopy

**Figure 10:**
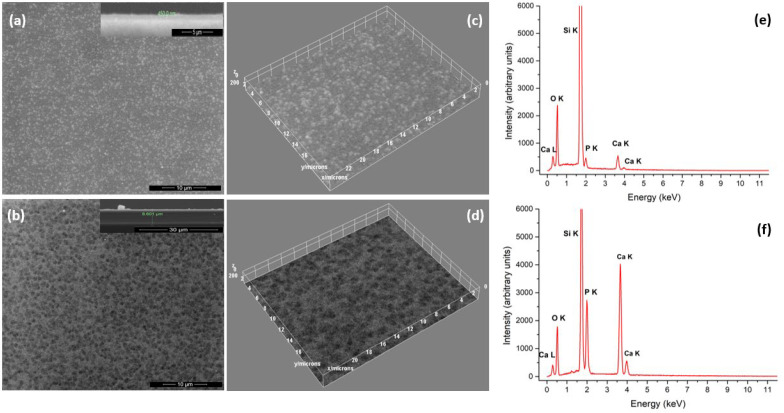
SEM images at 50 W (**a**) and 150 W (**b**), 3D images generated using ImageJ software at 50 W (**c**) and 150 W (**d**), and EDS spectra at 50 W (**e**) and 150 W (**f**) of CaP_PMMA layers.

The morphology of the surfaces of CaP_PMMA layers deposited on Si substrates are presented in Figure 10. The different features and their dependence on the rf power that modify the T_e_, n_e_ and n_i_ at the substrate holder position can be noticed in Figure 10a,b. Figure 10c,d presents the 3D plot profiles of the surfaces of CaP_PMMA layers shown in Figure 10a,b.

On the surface of the layers produced at 50 W rf power, some circular grain structures, uniformly distributed, are grown. The grain size has an average value of about 550 nm. This process could be attributed to the coagulation of the PMMA molecules after reaching the substrate surface.

The synthesis of polymer nanoparticles in plasma has been reported by multiple publications [10]. The polymer particles are formed in a three-step polymerization process: nucleation, coagulation, and growth of the molecular fragments extracted from the sputtering target. The nucleation and coagulation stages of polymer vapors into nanometer size particles are dependent on the presence of negative charges [59], such as electrons whose mobility is higher than those of positive ions. Therefore, the electron density can affect the shapes and sizes of polymer nanoparticles grown on the surface of a coating.

The influence of the T_e_, n_e_ and n_i_ parameters measured at 150 W rf power on the surface morphology is revealed in Figure 10b,d. It is dominated by the formation of flat texturized structures with sizes ranging between 600 and 1000 nm. The flatness of the structures on the range of hundreds of nanometers can be attributed also to the surface bombardment with more energetic species (mainly ions) that either deliver their energy through deposition or resputter the amorphous growing film. The ion density at the substrate holder increases at 150 W comparative to 50 W rf power, while the electron density decreases down to 3.1 × 10^14^ m^−3^.

Thereby, we can conclude that the morphology of the layer surface that contains PMMA molecules depends strongly on the ion and electron densities during the deposition processes.

The EDS spectra from Figure 10e,f reveal the chemical elements present in the layers, namely Ca, P, O, and Si coming from the substrate. The C K peak from 0.277 keV overlaps with the Ca L peak from 0.341 KeV and is not clearly evidenced in the EDS spectra. The lower intensity of the O K peak in the CaP_PMMA_150 sample is in accordance with the XPS and FTIR data that indicate a lowering of PMMA content. There are some studies that associated the reduced O content in a plasma polymer layer to the formation of inter-linked structures [26]. The intensities of Ca K and P K lines increase a few times at 150 rf power (see Figure 10f). It is suggested that a thicker CaP_PMMA layer is formed in accordance with deposition rate measurements and cross-section profiles inserted as captioned in the upper right side of Figure 10a,b. Due to the resputtering of the growing film mainly by negative oxygen ion bombardment, CaP_PMMA layer thicknesses are lower than the one indicated by the deposition rate measurements.

The atomic ratio of Ca/P is about 1.72 in the layers generated at 50 W rf power and about 1.9 in the layers generated at 150 W rf power. This increased retention of Ca was previously associated to the formation of amorphous calcium oxides in the layer [60,61]. The resputtering of the P atoms leads also to a higher Ca/P ratio.

## 4. Conclusions

In this work we report the deposition of CaP_PMMA layers by radio-frequency magnetron sputtering technique on silicon substrates at two working powers: 50 and 150 W. The Langmuir probe measurements indicated an increase in the electron temperature and ion density, a decrease in the electron density, and a shift to lower energies of the f(ε) as the power was increased from 50 to 150 W. Thus, more electrons are involved in the dissociation process of PMMA and the collision rate is increased as more ions are generated. These highlights were confirmed by the OES spectrum at 150 W, in which the C_2_ Swan band, the CO/OH molecular bands, and the H_α_ atomic line were identified, allowing at the same time the understanding of amorphous CaP_PMMA layer growing processes. A lower deposition power of 50 W preserves better the molecular structure of the polymer and maintains a Ca/P ratio (1.72) closer to the stochiometric Ca/P ratio of HAp. At a higher working power, the polymer IR characteristic bands are reduced and the Ca/P ratio increases to 1.9, most probably due to an increase in the TTCP amount. Anyway, more C-C/C-H and C-OH/C-O-C polymeric bonds were found at the surface of the Cap_PMMA_150 layer. As the power and ion density increase, the polymer heats up and the grain-like morphology is replaced by worm-like structures due to layer wrinkling Texturized CaP_PMMA layer surfaces deposited on medical implants could enhance bonding with the tissue for future biomedical applications.

## Figures and Tables

**Table 1:** Electron temperature, electron density and ion density measured at different power and radial position to the center of plasma.

Power (W)	Position in the Plasma Bulk (mm)	*T_e_* (eV)	*n_e_* (m^−3^)	*n_i_* (m^−3^)
50	0	6.9	4.6 × 10^14^	1.2 × 10^17^
	20	6.7	5 × 10^14^	8.5 × 10^16^
150	0	7.4	3.1 × 10^14^	2.2 × 10^17^
	20	7.4	4.7 × 10^14^	9.2 × 10^16^

## Data Availability

The original contributions presented in this study are included in the article. Further inquiries can be directed to the corresponding author(s).

## Abbreviations

The following abbreviations are used in this manuscript:

HAp	Hydroxyapatite
CaPs	Calcium phosphates
PMMA	Poly(methyl methacrylate)
MMA	Methyl methacrylate
rf-MS	Radio-frequency magnetron sputtering
f(ε)	Electron energy distribution function
OES	Optical Emission Spectroscopy
FTIR	Fourier Transform Infrared Spectroscopy
SEM	Scanning Electron Microscopy
EDS	Energy-Dispersive X-Ray spectroscopy
rf	Radio frequency
TTCP	Tetra calcium phosphate
